# Adipose-Derived Stem Cells from Type 2 Diabetic Rats Retain Positive Effects in a Rat Model of Erectile Dysfunction

**DOI:** 10.3390/ijms23031692

**Published:** 2022-02-01

**Authors:** Marlene Louise Quaade, Pratibha Dhumale, Simon Gabriel Comerma Steffensen, Hans Christian Beck, Eva Bang Harvald, Charlotte Harken Jensen, Lars Lund, Ditte Caroline Andersen, Søren Paludan Sheikh

**Affiliations:** 1 Laboratory of Molecular and Cellular Cardiology, Department of Clinical Biochemistry and Pharmacology, Odense University Hospital, 5000 Odense, Denmark; marlenelc85@gmail.com (M.L.Q.); pdhumale@health.sdu.dk (P.D.); ebhar@health.sdu.dk (E.B.H.); charken@health.sdu.dk (C.H.J.); dandersen@health.sdu.dk (D.C.A.); 2 Department of Cardiovascular and Renal Research, Institute of Molecular Medicine, University of Southern Denmark, 5000 Odense, Denmark; lars.lund@rsyd.dk; 3 Institute of Clinical Research, University of Southern Denmark, 5000 Odense, Denmark; hans.christian.beck@rsyd.dk; 4 Department of Biomedicine, Aarhus University, 8000 Aarhus, Denmark; simoncomerma@biomed.au.dk; 5 Department of Biomedical Sciences/Animal Physiology, Faculty of Veterinary, Central University of Venezuela, Maracay, Aragua 2105, Venezuela; 6 Centre for Clinical Proteomics, Department of Clinical Biochemistry and Pharmacology, Odense University Hospital, 5000 Odense, Denmark; 7 Department of Urology, Odense University Hospital, 5000 Odense, Denmark

**Keywords:** erectile dysfunction, adipose derived stem/stromal cells, type 2 diabetes mellitus, bilateral nerve crush injury

## Abstract

Erectile dysfunction is a common complication associated with type 2 diabetes mellitus (T2DM) and after prostatectomy in relation to cancer. The regenerative effect of cultured adipose-derived stem cells (ASCs) for ED therapy has been documented in multiple preclinical trials as well as in recent Pase 1 trials in humans. However, some studies indicate that diabetes negatively affects the mesenchymal stem cell pool, implying that ASCs from T2DM patients could have impaired regenerative capacity. Here, we directly compared ASCs from age-matched diabetic Goto–Kakizaki (ASC_GK_) and non-diabetic wild type rats (ASC_WT_) with regard to their phenotypes, proteomes and ability to rescue ED in normal rats. Despite ASC_GK_ exhibiting a slightly lower proliferation rate, ASC_GK_ and ASC_WT_ proteomes were more or less identical, and after injections to corpus cavernosum they were equally efficient in restoring erectile function in a rat ED model entailing bilateral nerve crush injury. Moreover, molecular analysis of the corpus cavernosum tissue revealed that both ASC_GK_ and ASC_WT_ treated rats had increased induction of genes involved in recovering endothelial function. Thus, our finding argues that T2DM does not appear to be a limiting factor for autologous adipose stem cell therapy when correcting for ED.

## 1. Introduction

The epidemic-like increasing prevalence of type 2 diabetes mellitus (T2DM) is associated with numerous complications and reduced life quality for the patients, in addition to substantial societal economic costs related to treatment [1,2]. Diabetic men rank erectile dysfunction (ED) as their third-worst complication [3] and approximately 75% of the patients suffer from ED [4,5]. In addition, diabetics experience ED earlier in life and in a more severe form than otherwise seen in the healthy male population [6]. It has profound negative effects on the life quality of not only the patients but also their partners. Endothelial dysfunction with reduced nitric oxide (NO) release and impaired neurotransmission has been implied as the underlying pathological mechanisms that cause ED in diabetic men [4,5]. Likewise, and independent of diabetes, a significant number of men experience ED after prostatectomy in relation to prostate cancer. As in ED in diabetic men, the underlying cause relates to the neural system, where the nerves are damaged during prostatectomy. Conventional treatment i.e., oral dosages of phosphodiesterase 5 inhibitors are often ineffective, because their therapeutic efficacy is based on endothelial cells and nitric oxide signaling pathways [7]. Thus, there is a high demand for new alternative therapies for patients with neurogenic ED.

Stem cell therapy has long been considered a promising approach for correcting ED and the efficacy has been tested in different animal models [8,9]. Kim et al. have reviewed the different sources from which stem cells have been derived and used for treatment of ED, i.e., bone marrow, adipose tissue, umbilical cord blood, mononuclear blood cells and neural crest stem cells [10]. Compared to other autologous tissue sources including bone marrow, adipose tissue has the advantage that it can be easily obtained with less discomfort for the patient and most importantly, it contains a large number of progenitor cells [3,11,12]. Upon enzymatic digestion of adipose tissue, the freshly isolated heterogeneous population of cells obtained is referred to as the stromal vascular fraction (SVF). When culturing SVF in a defined media it forms a homogenous population of mesenchymal stem cells dubbed adipose stem cells (ASCs). ASCs are multipotent progenitor cells that can self-renew, differentiate into other cell types [13,14], and secrete multiple growth factors and cytokines resulting in regenerative effects in various diseases including ED [15,16,17,18]. We have previously demonstrated safety and potential therapeutic efficacy of freshly isolated SVF in a Phase I clinical trial treating ED patients following radical prostatectomy [19]. This underscores the potential benefit of ASCs for correcting ED.

However, previous studies have suggested that Diabetes Mellitus (DM) impairs the function of the progenitor cells themselves, including ASCs, and they have been reported to exhibit decreased proliferation rates, growth factor secretion and angiogenic potential [20,21,22,23,24]. However, detailed in vitro and in vivo studies investigating the regenerative potential of diabetic and non-diabetic ASCs in relation to correcting ED remains to be determined.

We therefore set out to directly determine and compare the regenerative potential of diabetic and healthy ASCs in a rat model of neurogenic ED.

## 2. Results

### 2.1. Diabetic and Non-Diabetic ASCs Are Similar during In Vitro Conditions

**Figure 1:**
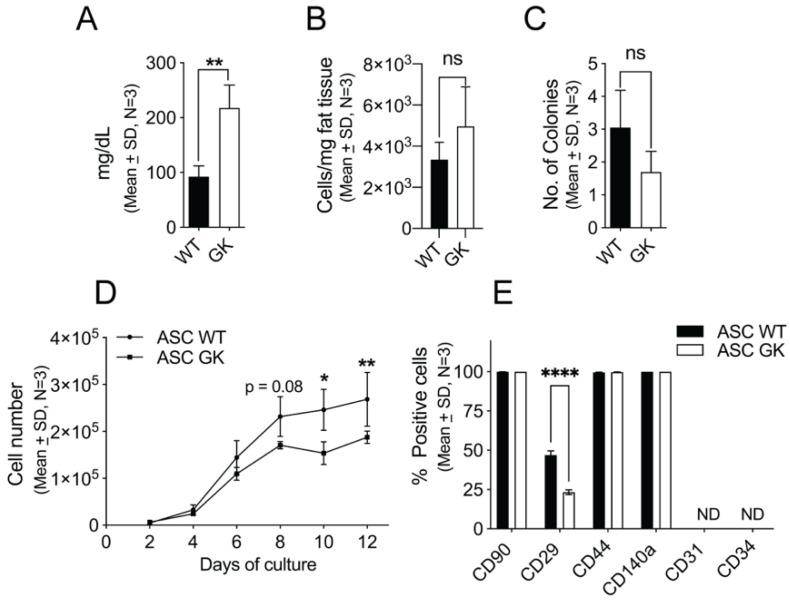
Characterization of ASC from WT and GK rats. (**A**) Blood glucose levels were measured in tail vein blood from 17-week-old wildtype (WT) and diabetic Goto–Kakizaki (GK) rats to validate their non-diabetic/diabetic status, respectively. (**B**) The primary yield of nucleated cells in SVF from SAT of WT and GK expressed per mg of adipose tissue (cells/mg of tissue). (**C**) The frequency of stromal progenitors in the original SVF was determined by CFU-F assays of parent SVF_WT_ and SVF_GK_ cells. (**D**) Proliferation of ASC_WT_ and ASC_GK_ were followed by cell number assessment after 2, 4, 6, 8, 10 and 12 days of culture. (**E**) The presence of well-known mesenchymal stem cell surface markers of ASC_WT_ and ASC_GK_ cells were analyzed by flow cytometry. For each marker, the percentage of positive cells was obtained by gating using the appropriate isotype control. CD31 and CD34 expression were not detected (ND). Not significant (ns). Data are shown as mean ± SD, *n* = 3 and statistical significance was tested using a nonparametric Mann–Whitney test in (**A**–**C**) and by two-way ANOVA test with Sidak’s multiple comparison test in (**D**,**E**). * *p* < 0.001, ** *p* < 0.0001.

**Figure 2:**
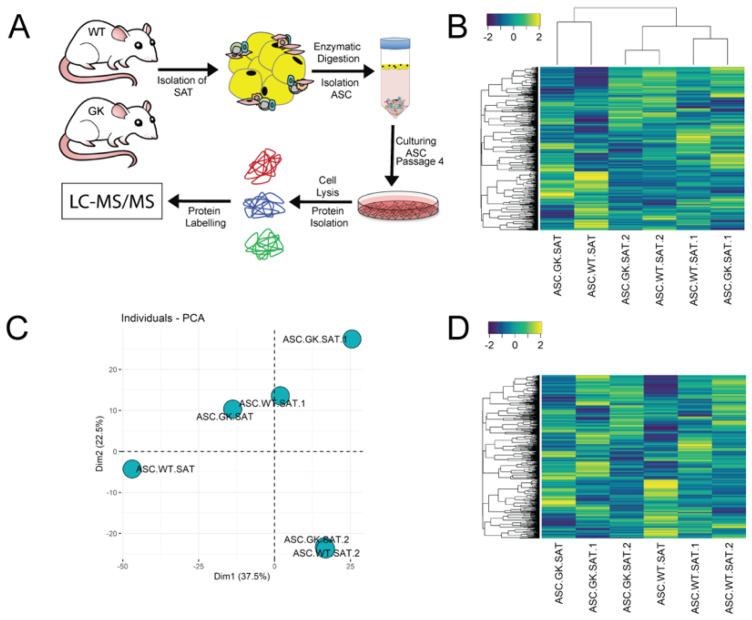
Quantitative proteomic analysis on ASCs from WT and GK rats. (**A**) Schematic representation of the TMT proteomic study. (**B**,**C**) Cluster analysis of proteins expressed in ASC_WT_ (*n* = 3) and ASC_GK_ (*n* = 3) with heatmap analysis was performed in “R”. Heatmap (**B**) and PCA plot (**C**) showing no association between samples. (**D**) Heatmap with no dendrogram clustering of samples. The legend color bar in (**B**,**D**) indicates the relation between scaled ratios and colors.

To determine whether the type 2 diabetic state has an effect on the regenerative potential of ASCs, we used the non-obese type 2 diabetic (GK) rats along with normal age-matched WT rats for isolation of donor ASCs. Initially, we isolated SVFs from subcutaneous adipose tissue (SAT, inguinal fat pads) and cultured them to obtain ASCs. As expected, the ASC cultures were relatively more homogenous than their parent SVF and consisted of CD45−/CD31−/CD34−/CD44+/CD90+ cells (Figure S1A) in agreement with others [25,26]. Moreover, as expected ASCs exhibited an adipogenic-, chondrogenic- and osteogenic differentiation potential as well as the ability to form capillary-like structures, when stimulated (Figure S1B). The established approach was then used in a new series of experiments on SAT from WT and GK rats. Prior to fat harvest, blood glucose levels were measured and found to be significantly elevated in GK rats as compared to WT rats (217.9 ± 41.8 and 92.3 ± 19.7 mg/dL, (*n* = 3), *p* = 0.0092) (Figure 1A) confirming the diabetic state of the GK rats and underscoring the study design. We then obtained ASCs from GK- (ASC_GK_) and WT- (ASC_WT_) rats by harvesting SAT followed by SVF isolation as established above. No significant difference was observed in the yield of nucleated primary cells (SAT_GK_: 3344 ± 846 cells/mg tissue and SAT_WT_: 4967 ± 1915 cells/mg tissue, *t*-test (*n* = 3), *p* = 0.4) between wildtype and diabetic rats (Figure 1B). Similarly, the frequency of stromal progenitor cells in the SVF from WT and GK rats were comparable as determined by CFU-F assays as in general (Figure 1C). Upon 12 days culture, both ASC_GK_ and ASC_WT_ showed sigmoid-shaped proliferation curves as expected. However, ASC_GK_ displayed a slightly less steep growth between day 4-8 of culture resulting in a final lower number of ASCs_GK_ as compared to ASCs_WT_ at day 10 (*p* = 0.03) and day 12 (*p* < 0.0001) (Figure 1D). Flow cytometric analysis of classic mesenchymal stem cell markers on ASC_WT_ and ASC_GK_ revealed that CD90, CD44, and CD140a were expressed in all ASCs independent of diabetic status whereas CD31 and CD34 were absent in agreement with our initial setup (Figure 1D). We did observe Itgb1 (CD29) to be expressed in 47.0 ± 2.7% of ASC_WT_ and 23.3 ± 1.6% of ASC_GK_ (*p* < 0.0001) (Figure 1B) likely reflected heterogeneity for CD29 expression in ASCs in general. To enable a more detailed comparison of ASC_GK_ and ASC_WT_, we exploited quantitative mass spectrometry of the ASCs themselves (Figure 2A). We identified a total of 2144 proteins across all samples, where 1601 of them were present in all six samples. Ingenuity analysis confirmed that identified proteins originated from all parts of the cell (Figure S2) validating the approach used. However, clustering analysis (Figure 2B) and principal component analysis PCA (Figure 2C) plotting of all 6 samples showed no association between samples and the diabetic state of origin. With a False Discovery Rate (FDR) of 5%, we did not detect significant differences in any of the identified proteins between ASC_WT_ and ASC_GK_ (data not shown). This similarity was also reflected by the appearance of heatmap scattering despite ordering of samples into WT and diabetic origins (Figure 2D). Together these data thus suggest that the diabetic state of the GK rats does not significantly affect the overall derived phenotype of ASCs. Since the proteome data includes several IDs with missing values (305 of 1906), we speculate if there would be any change between diabetic and non-diabetic ASCs when including these IDs. To that end, estimated values were calculated for all missing values using the R package missMDA. As for the analysis above, this did not result in any difference between diabetic and non-diabetic ASCs (Figure S4).

### 2.2. The Diabetic State Does Not Impair the Effect of ASCs on Erectile Recovery In Vivo

**Figure 3:**
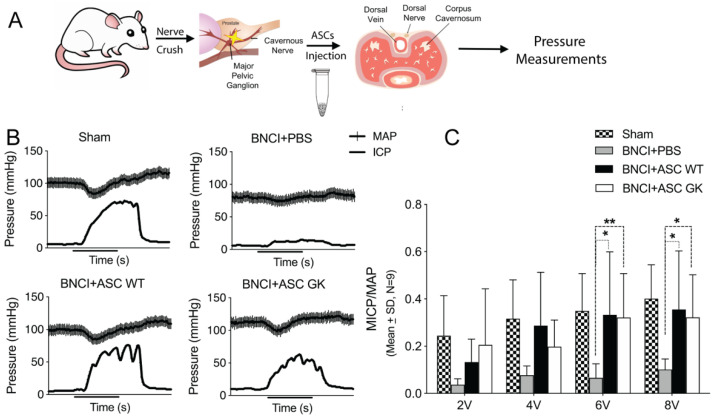
Assessment of erectile function following ASC_WT_ or ASC_GK_ treatment. (**A**) Schematic representation of the in vivo experiment. Rats were subjected to bilateral nerve crush injury (BNCI) followed by penile injection of vehicle (PBS) or ASC_WT_ or ASC_GK_ and compared with sham controls (*n* = 9 in each group). 28 days after surgery the erectile function was evaluated by intracavernosal pressure (ICP) (mmHg) recordings during electro-stimulation of 2, 4, 6 or 8V. (**B**) Representative ICP and mean arterial pressure (MAP, mmHg) traces in response to 8V electro-stimulation for 50 s (marked by a line below the *x*-axis) in Sham, BNCI+vehicle, BNCI+ASC_WT_ and BNCI+ASC_GK_ treated rats are shown. (**C**) The maximum ICP increase (MICP) was normalized to MAP by calculating the MICP/MAP ratio in order to compare erectile function between animals. Data are represented as mean ± SD, and statistical significance was tested by mixed-effect model (REML) with Tukey’s multiple comparison test. * *p* < 0.05, ** *p* < 0.01.

Although ASCs of healthy and diabetic donor origin appear similar in vitro, confirmation of functional ED recovery is required to evaluate the therapeutic potential of ASCs with a diabetic origin. To simulate erectile dysfunction in rats, we performed bilateral cavernous nerve crush injury in WT rats (BNCI; Figure 3A). We then injected 10^6^ ASCs_WT_ or ASCs_GK_ into the penis of nine rats in each group and quantified the erectile function at day 28 post injury (Figure 3A). To obtain a robust functional output, we performed a series of electro-stimulations (2, 4, 6, and 8V) to the cavernous nerve, where the mean arterial pressure (MAP) and simultaneous recording of the ICP in the corpus cavernosum of the penis were obtained. As previously described [27] we determined peak ICP, maximum ICP (MICP) and MICP/MAP ratio (Figure 3B) to more firmly determine erectile function. This model has been widely used to evaluate stem cell therapy for ED (see additional references) [28,29,30]. At day 28, the erectile function was substantially reduced in the vehicle group as compared to the sham group (Figure 3C and Figure S3A,B) thus validating the BNCI model. However, injections of both ASCs _WT_ and ASCs _GK_ rescued erectile function as compared to vehicle control 28 days after BNCI (Figure 3C and Figure S3A,B). Importantly, no significant differences were observed between ASCs _WT_ and ASCs _GK_ in erectile functional recovery at any voltages (2 V, 4 V, 6 V: *p* > 0.999, 8 V: *p* = 0.823). These data thus underscore that the presence of diabetes in the ASC donor individual does not impair the therapeutic potential of ASCs for correcting erectile dysfunction.

### 2.3. Independent of Donor Diabetic State, ASC Treatment Induces Endothelial Repair

**Figure 4:**
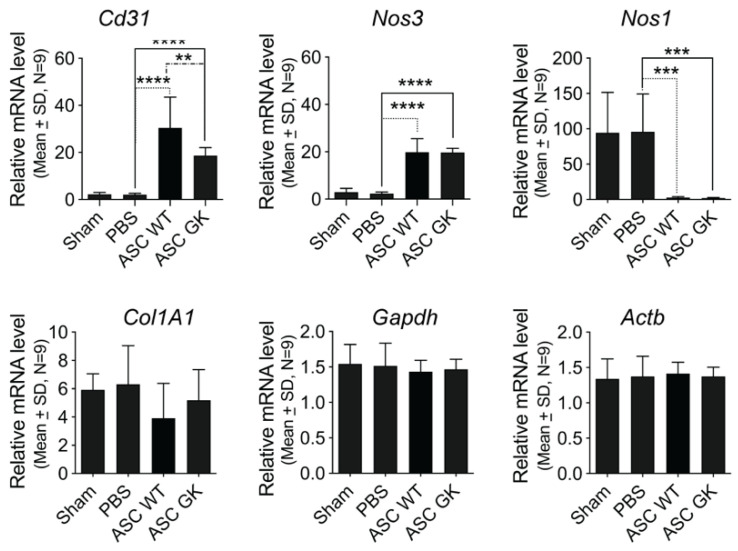
ASCs derived from both WT and GK rats induce endothelial gene expression in vivo: mRNA expression of endothelial markers *Cd31 (Pecam1*) and *Nos3* (*eNOS*), neuronal marker *Nos1* (*nNOS*) and fibrotic marker *Col1a1* (*Pro-collagen 1*) were analyzed by qRT-PCR of corpus cavernous tissue from rats as indicated by treatment group (*n* = 9 in each). All data were normalized to two stably expressed reference genes, *Gapdh* and *Actb* (*β-actin*). Data is represented as mean ± SD, and statistical significance was tested by ordinary one-way ANOVA with Tukey’s multiple comparison test. ** *p* < 0.01, *** *p* < 0.001, **** *p* < 0.0001.

We finally assessed the underlying mechanism for ASC mediated repair in both ASCs _WT_ and ASCs _GK_ treated rats by analyzing the corpus cavernosum after termination of the study. Quantifying endothelial and fibrosis markers, we found that endothelial repair was markedly enhanced in both ASCs _WT_ and ASCs _GK_ treated rats as compared to sham and PBS injected rats (Figure 4). As such, expression of the endothelial marker *Cd31* was significantly elevated after treatment with both ASC_WT_ and ASC_GK_ as compared to vehicle control (30.4 ± 13.1 and 18.7 ± 3.4 versus 2.1 ± 0.6, **** *p* = 0.0004). Likewise, endothelial nitric oxide synthase (*eNOS or Nos3*) expression was significantly increased in both ASCs treated groups (ASC_WT_; 19.9 ± 5.7, **** *p* < 0.0001 and ASC_GK_; 19.7 ± 1.8, **** *p* < 0.0001) compared to the vehicle control (2.3 ± 0.7). On the opposite, expression of neuronal nitric oxide synthase (*nNOS or Nos1*) was significantly decreased in the ASC treated groups (ASC_WT_; 2.6 ± 1.5, *** *p* = 0.001 and ASC_GK_; 2.2 ± 0.9, *** *p* < 0.0003) as compared to the vehicle control (95.85 ± 53.3). No difference was observed between ASC_WT_ and ASC_GK_ in the expression of either *eNOS* or *nNOS*, but the ASC_WT_ population seemed slightly superior to ASC_GK_ to induce *Cd31* expression (** *p* = 0.0041) (Figure 4). No difference was observed in the expression of the fibrotic marker *Pro-collagen 1* (*Col1A1*) between treated rat groups.

These results thus indicate that ASC treatment, at least partly, corrects the ED phenotype by restoring the endothelium and the ability to release NO in the corpus cavernousum. Moreover, these data further support that the diabetic ASC donor origin does not overall affect the regenerative ability of the ASCs.

## 3. Discussion

The therapeutic effect of ASCs has been widely studied in regenerative medicine in both animals and clinical trials [31]. However, a considerable proportion of patients that could benefit from adipose stem cell therapy are diabetic which prompted us to investigate whether autologous ASCs from diabetic individuals retain the same regenerative capacity for ED repair. Overall, we found no apparent difference between ASC of diabetic and non-diabetic origin, and they were equally effective in ED recovery in vivo.

We chose to use GK rats, a non-obese and spontaneous (genetic) T2DM experimental model, that exhibits defective pancreatic β cell mass and function, similar to human diabetic patients [32]. While we did not observe any difference in the yield of SVF cells and numbers of CFUs between GK and WT rats, others have recently reported both parameters to be reduced in diabetic patients [24]. We did though, recognize a slightly compromised ASC proliferation rate but as other groups have reported results varying from no difference to lower and higher proliferation potential of “diabetic” ASCs [23,24,33,34], this suggests that study design and use of methods may likely influence the outcome. Our study design isolating wild type and diabetic adipose-derived cells and testing their effects in wildtype rats have not previously been employed precluding direct comparisons with the literature. However, our principal conclusion is strongly supported by work in mice from Wang et al. showing that ‘diabetic’ stem cells were less proliferative but retained biological activity albeit on diabetes, while the effect on ED was not investigated. Thus, ASCs from high-fat diet and streptozotocin-induced type 2 diabetes had inferior proliferative capacity compared to cells from healthy controls, improved insulin sensitivity and less β cell death was seen in T2D mice receiving mesenchymal stem cell therapy [35].

Diabetes is a multifaceted disease and has been reported to alter the expression of many genes and related proteins [36,37], but to our knowledge comparative proteome analysis of ASCs remains to be performed. Herein, we used quantitative mass spectrometry of ASCs, but did not observe any significant differences between the proteomes of diabetic and non-diabetic ASCs. However, since the quantitative method of mass spectrometry used herein only identifies the most abundantly expressed proteins, we cannot exclude that less abundant proteins such as transcription factors may differ between diabetic and non-diabetic ASCs. Other proteomic studies of whole epididymal VAT from diabetic or insulin-resistant animals and patients have indeed been demonstrated to exhibit altered abundances in many proteins particularly those involved in lipid metabolism and inflammation [38,39]. Whether such discrepancies are explained by the methods used or by proteomes of whole fat and the fat-derived ASCs being different, we can only speculate. Yet, by our approach, no apparent difference was observed between any proteins identified in the mass spectrometry of diabetic and non-diabetic ASCs.

Whereas several in vitro studies in recent years have tested similarities and differences between ASCs of diabetic and non-diabetic origins, in vivo studies are limited and lacking with respect to ED repair. We evaluated the regenerative abilities of ASCs to correct ED in the BNCI model [40]. Although the pathogenesis of human diabetes is multimodal, the rat ED model at least includes a nerve lesion, penile fibrosis and its wide use as a pre-clinical model has contributed to significant advancement in the study of ED [1,19,40,41,42]. This model is generally considered valid for ED patients, and we were able to confirm earlier reports showing recovery of erectile function by injecting a single bolus of ASCs and most importantly, found that the ASC_WT_ and ASC_GK_ were equally potent to improve erectile recovery (up to 89% of sham). This is in agreement with another in vivo setup of ASC function, where Gu et al. showed similar functional improvements in an ischemic flap mouse model using ASCs from diabetic and non-diabetic human subjects [43]. By contrast, Rennert et al. reported that the diabetic state of mouse SVF impairs their neovascularization and wound healing capacity in vivo [22]. Yet, SVF is quite different from ASCs as also revealed herein and may explain the differences. Indeed, impairment of endothelial function, decreased levels of growth factors and cytokines along with fibrosis have been reported in the corpus cavernosum of diabetic patients and also in preclinical models [44,45]. In this respect, ASCs have been demonstrated by others to recover endothelium function and vascularization through elevating VEGF and eNOS levels in corpus cavernosum of ED rat models [46]. Our data also suggest that both ASC_GK_ and ASC_WT_ induce vascular repair in the corpus cavernosum after treatment, which may explain the beneficial functional outcome. In this regard, it is important to consider that the penile erection mechanism is based on smooth muscle relaxation induced by nitric oxide (NO) which in turn is generated by nNOS in neurons and by eNOS in endothelial cells. On the other hand, we observed an upregulation of *eNOS*, there was a concomitant downregulation of *nNOS* in both groups as compared to sham and vehicle control rats. This indicates that endothelial repair rather than neuronal regeneration is responsible for the erectile recovery effect seen herein. This corroborates previous findings where ASC mediated eNOS improves the microenvironment of corpus cavernous tissue [10,47]. The slight difference in *Cd31* expression between ASC_GK_ and ASC_WT_ treated animals seems insignificant, since the functional outcome was similar between the groups. However, we realize that these findings are based on an animal model that may not fully replicate the complex nature of human sexual function.

Even though an allogenic treatment might be available in the future, the advantages of an autologous approach likely include less immune reaction, easier regulatory approval and public acceptance thus a faster route to market availability. Thus, overall, our findings suggest that generally, ASCs from spontaneous type 2 diabetes GK rats are similar to those of non-diabetic rats. In perspective, this indicates that autologous ASC therapy is not limited by the diabetic state of the ASC origin. However, whether the diabetic state and microenvironment of the patient or animal itself may alter the effect of diabetic ASCs on ED recovery remains to be determined in the future.

## 4. Material and Methods

### 4.1. Animals for Stem Cell Harvest

Spontaneous type 2 diabetic Goto–Kakizaki (GK) rats and Wistar (WT) rats, all male, were purchased from Taconic Europe. GK rats were fed NIH31-M rodent diet (Brogaarden, Lynge, Denmark) by recommendation from Taconic. All other animals were fed regular altromin 1324 (Brogaarden). At 18 weeks of age, animals were sacrificed to harvest inguinal adipose tissue for SVF isolation and tissue culture. According to the manufacturer, the GK rats spontaneously develop type 2 diabetes at 14–16 weeks of age. After five hours of fasting, blood glucose levels were evaluated in tail vein blood at 17 weeks of age by using the OneTouch, Ultra Easy instrument (Mediq, Brøndby, Denmark).

### 4.2. Cell Isolation and Culturing

For the generation of cultured ASCs for in vivo transplantation, GK rats and WT rats were euthanized using carbon dioxide, and the inguinal fat pads were collected. Adipose tissue was minced and enzymatically digested with collagenase (0.86 U/mL Collagenase NB 4 Standard Grade, Serva, Germany). Following red blood cell lysis and filtration, the SVFs were isolated and counted using a NucleoCounter NC-200 instrument (ChemoMetec, Allerød, Denmark). SVF aliquots were either fixed immediately for flow cytometry, seeded for colony-forming-unit fibroblast CFU-F assay or cultured for in vitro expansion to achieve ASCs.

For expanding cell number and increase homogeneity, 10^6^ SVF were seeded in T75 cell culture flasks in growth medium (DMEM/1.0 g/L glucose/25 mM HEPES supplemented with 4 mM Ultraglutamine/10% Fetal Bovine Serum (FBS) (all products from Lonza, UK)/1% PS) and cultured at 37 °C and 5% CO_2_. Non-adherent cells were removed after 24 h while refreshing media. Hereafter, the growth medium was changed every third day, and the cells passaged until number 4 with approximately 80% of confluence.

### 4.3. Colony Forming Unit Assay

Freshly isolated SVF were seeded in triplicates in 6-well plates at a density of 100 cells/cm^2^ and cultured for twelve days. Following a PBS wash the cells were fixed in 4% neutral buffered formalin (NBF) for 20 min, at room temperature RT, washed twice in PBS and stored at 4 °C until staining. For staining, the cells were rinsed in tap water and incubated 5 min, at RT with Mayer’s hematoxylin. Cells were washed and plates were left inverted to dry overnight. The number of colonies (>30 cells) was determined using a stereo microscope (Leica M80).

### 4.4. Flow Cytometry

Freshly isolated SVF or Cultured ASCs (passage 4) were washed in Hank’s balanced salt solution (HBSS)/1% PS/5% FBS and fixed in HBSS/5% FBS/1% PS/1% NBF overnight at 4 °C. The cells were then washed twice and stored in HBSS/1%PS/5% FBS/0.05% sodium-azide at 4 °C until analysis. Fixed cells were washed in HBSS/1% PS/5% FBS and incubated 60 min with primary antibodies on ice while shaking. After, two washes the cells were incubated 30 min with secondary antibodies on ice while shaking and finally washed twice. Data acquisition and analysis were obtained using a FACSCalibur instrument (Becton Dickinson, 2150 Commerce Dr, San Jose, CA, USA) and FlowJo 10.0.6 software (Tree Star Inc., OR, USA), respectively. Primary antibodies were specific for rat CD45, CD90, CD44 (BD bioscience, 554,875 (1:100), 554,895 (1:50); and 554,869 (1:100), respectively), and from other companies CD29 (abcam, ab52971 (1:100)), CD34 (R&D Systems, MN, USA, AF6518 (1:72)), PDGFRα (cell signaling, 3164 (1:200) and CD31 (Santa Cruz, CA, USA, sc-1506 (1:100)). Isotypes included sheep IgG (R&D systems, 5-001-A), rabbit polyclonal IgG, rabbit monoclonal IgG (abcam, ab37415, ab125938), mouse IgG2a,k, and mouse IgG1,k (Sigma-Aldrich, Vandtårnsvej 62A, Søborg Denmark M 5409, M 5284). Alexa 488 or 647 conjugated secondary donkey antibodies specific for rabbit IgG, mouse IgG, sheep IgG (all purchased at Invitrogen, 3 Fountain Drive Inchinnan Business Park, Paisley, UK, 1:200) were used for visualization.

### 4.5. Differentiation

StemPro Differentiation Kits (Gibco, Life Technologies, Scientific 151 Brook Drive, Milton Park Abingdon, UK) were used to verify the differentiation capacity of the cultured ASCs. Differentiation protocols were executed according to the manufacturer’s recommendations. Briefly, passage 4 ASCs were incubated with an Adipogenesis Differentiation Kit for 19 days, Osteogenesis Differentiation Kit for 12 days and a Chondrogenesis Differentiation Kit for 14 days. Adipogenic differentiation was confirmed using Oil Red O (Sigma-Aldrich, Vandtårnsvej 62A, Søborg Denmark) staining. Osteogenesis was confirmed using alkaline phosphatase detection kit (EMD Millipore, Darmstadt, Germany, #SCR004) and finally, chondrogenic differentiation was confirmed by alcian blue staining (Fagron Nordic A/S, Kigkurren 8M, Copenhagen, Denmark). Undifferentiated cells were used as negative controls. The cells were photographed using an inverted microscope (Leica DMI4000B).

Additionally, endothelial differentiation was induced in passage 4 ASCs by incubation in endothelial cell growth basal medium (EBM-2) (Lonza, CC-3156) supplemented with EGM-2MV SingleQuots Kit (Lonza, CC-4147)) for 13 days. Cells were harvested using TrypLE and cells were seeded at a density of 24,200 cells/cm^2^ in 24-well plates coated with 200 µL growth factor reduced Matrigel (Corning, NY, USA, #354230). Following 17 h of culture in EGM tube-like structures were examined by phase microscopy using an inverted microscope (Leica DMI4000B).

### 4.6. Proliferation Assay

Passage 0 ASC stock was cultured and passaged before being seeded as passage 3 in 6-well plates with 500 cells/cm^2^. Growth medium was changed every second day. Three wells from each animal were harvested for cells using TrypLE (Thermo Fisher Scientific, Scientific 151 Brook Drive, Milton Park Abingdon, UK) for 7 min at 37 °C, at each of the time points: 2, 4, 6, 8, 10, 12 days. Cell counting was conducted in triplicates by using hemocytometer and trypan blue (Gibco).

### 4.7. Animals Model of Erectile Dysfunction and In Vivo Cell Transplantation

37 Sprague Dawley (SD) rats (12 weeks old), all male, were purchased from Taconic Europe and all rats were randomly divided into four groups; (A) Sham Control (*n* = 10), (B) rats without treatment (sterile PBS, *n* = 9), rats treated with ASC WT (ASC WT, *n* = 9) and rats treated with ASC GK (ASC GK, *n* = 9). Rats were anaesthetized subcutaneously (SC) using a mixture of hypnorm (236 µg/kg fentanyl and 7.5 mg/kg fluanisone) and midazolam (3.75 mg/kg), placed in a supine position and a midline incision was made along linea alba. For all animals, the prostate, the major pelvic ganglion (MPG) and cavernous nerve (CN) were identified on both sides. In the sham group, no further surgery was performed. In the remaining groups, the CNs were crushed with the same force, for exactly 2 min. using the tips of a dedicated needle holder [33].

Then the penis was exposed through the lower abdominal incision, a tourniquet was placed at the base of the penis and 10^6^ ASC from WT or GK rats in 200 µL PBS was injected into the corpus cavernosum at the mid-penile level. To prevent liquid backflow and to allow the cells to settle, pressure was applied to the injection site for 1 min and the tourniquet was removed 2 min following injection. The wound was then closed in two layers and Temgesic (0.04 mg/kg SC) was administered subsequently. Postsurgical analgesia was administered by voluntary ingestion of Nutella containing Temgesic (0.3 mg/kg).

### 4.8. In Vivo Evaluation of Erectile Function

Erectile function was evaluated after 28 days of injury and ASC (WT or GK) transplantation by intracavernous pressure (ICP) response to CN electrostimulation. Rats were anesthetized by intraperitoneal injection of a mixture of ketamine (100 mg/kg) and xylazine (10 mg/kg). The left carotid artery was identified and cannulated with a heparinized (100 U/mL) fine bore polythene catheter (PE, 0.58 mm ID × 0.96 mm OD, Portex, Smiths Medical, Kent, UK) connected to a pressure transducer (Utah Medical Products, Midvale, UT, USA) for continuous measurement of systemic blood pressure (mean arterial pressure (MAP)). The abdomen was opened with a midline incision and the prostate, the MPG and the CN were identified. The testicles were relocated to the upper abdomen to ease the access to the crus of the penis. The penis was exposed and dissection continued to expose the crus under the ischiocavernous muscle, which was transected. A bipolar hook electrode was placed on the CN distal to the crush injury. The crus of penis was then cannulated with a heparinized 25G needle with a PE-50 catheter (Portex, Smiths Medical) connected to a pressure transducer for measurement of ICP. The CN electrostimulations were performed using a custom-made stimulator. In accordance with the majority of similar studies, the stimulus parameters were 2, 4, 6 and 8V, 20 Hz, pulse width of 0.5 and duration of 50 s, with a minimum of 5 min rest interval between stimulations [41]. The CN and the area were kept dry prior to stimulations. Following every stimulation series, the CN was transected distal to the electrode and a stimulation of 8V was applied to check for non-CN-mediated retrograde ICP response. In the case of such a response, the stimulation series was rejected as a false positive and the contralateral CN was subjected to a new series of electro-stimulations. All animal experiments were approved by the Danish Council for Supervision with Experimental Animals (#2013-15-2934-00877). All data was collected by Labview (National Instruments, Austin, TX, USA) and analyzed using GraphPad Prism (9.0d Mac OS X, 2365 Northside Dr., San Diego, CA, USA).

### 4.9. RNA Isolation and qRT-PCR

For RNA extraction and subsequent qRT-PCR analysis, the corpora cavernosum were dissected and rapidly frozen using dry ice. RNA isolation and relative quantitative reverse transcription-polymerase chain reaction (qRT-PCR) was used to examine relative gene expression in the corpus cavernosum. Accordingly, dissected corpus cavernosum samples were homogenized and the total RNA was extracted using a TriReagent protocol (Thermo Fisher Scientific, Scientific 151 Brook Drive, Milton Park Abingdon, UK). RNA purity and quantity was assessed by nanodrop (Nanodrop^®^ Technologies, Thermo Fisher Scientific, Scientific 151 Brook Drive, Milton Park Abingdon, UK) measurements. mRNA was reverse transcribed to cDNA using a High Capacity cDNA kit (Applied Biosystems, Thermo Fisher Scientific, Scientific 151 Brook Drive, Milton Park Abingdon, UK) and the qRT-PCR reaction was carried out using Power SYBR^®^ Green PCR kit (Applied Biosystems) and primers specific for *Cd31*, *Procollagen1*, *αSMA*, *eNOS*, *nNOS* (Primer sequences not shown). All qRT-PCR analyses were performed with the 7900HT Fast Real-Time PCR System (Applied Biosystems) instrument and data was normalized against stably expressed control genes, GAPDH and β-actin (Mean M = 0,431 and Mean CV = 0.150) using the qBase^+^ software.

### 4.10. Proteome Analysis

ASCs (Passage 4) obtained from inguinal adipose tissue from GK or WT rats were counted using the NucleoCounter instrument and 2 × 10^6^ ASCs were washed in PBS three times to avoid protein contamination from the FBS.

For protein isolation, cells were re-suspended in lysis buffer (10 mM EDTA, 300 mM NaCl, 0.2% Triton X100, 200 mM Tri-ethylammonium bicarbonate-TEAB, Complete mini protease inhibitor) and disrupted by sonication. Cell debris was pelleted by centrifugation (20,000× g for 60 min at 4 °C) and proteins were isolated by transferring the supernatant to 5 equivalents ice-cold acetone. Proteins were then reduced in the presence of 5 mM dithiothreitol (DTT) and incubated for 30 min at 50 °C followed by blocking of the reduced sulfhydryl groups with 15 mM iodoacetamide for 30 min in darkness. Trypsin (Promega, Madison, WI, USA) was added at protein:trypsin ratio of 50:1 *w*/*w*, followed by overnight incubation at 37 °C.

### 4.11. Stable Isotope Labeling of Protein Samples with TMT-10 Plex

A 10-µg fraction of the tryptic digest was collected for labeling with 10-plex TMT-kit (Thermo Scientific). The content of each TMT reagent vial was re-suspended in anhydrous ethanol, and a 40-µg sample was labeled. Labeled samples from each biological experiment (*n* = 3) were pooled in equal ratios, dried in a vacuum centrifuge, re-dissolved in 50 µL trifluoroacetic acid solution (0.1%), and purified loaded on a microcolumn packed with reversed phased material (equal *w*/*w* amounts of Poros R2 and Oligo R3 material) and fractionated into 7 fractions by high pH liquid chromatography virtually as previously described [48].

The Fractions were analyzed by RP-nanoLC-MS/MS analysis on an Orbitrap Eclipse mass spectrometer (Thermo Fisher Scientific) equipped with a nanoHPLC interface (Dionex UltiMate 3000 nano HPLC). Briefly, samples were separated using linear 49 min gradients (fractions 1–3) and 77 min linear gradients (fractions 4–7) ranging from 93% solvent A (0.1% formic acid) to 34% solvent B (80% acetonitrile/0.1% formic acid). Mass spectra of eluting peptides were acquired in positive ion mode applying automatic data-dependent switch between an Orbitrap survey MS scan in the mass range of 400–1200 *m*/*z* followed by peptide fragmentation applying a normalized collisional energy of 40% in a 3-s duty cycle. The automatic gain control (AGC) target was set to “250%” at a resolution of 60,000 at *m*/*z* 200, and 200,000 ions at a resolution of 50,000 at *m*/*z* 200 for MS/MS scans. Ion selection threshold for MS/MS analysis was set to 50,000 counts. Selected sequenced ions were dynamically excluded for 60 s. All raw data files were processed and quantified using Proteome Discoverer version 2.4 (Thermo Scientific) also as previously described [49] with the exception that the database search was restricted to the rat database instead of humans. For principal component analysis (PCA), all missing values were removed (a total of 1601 proteins were used) prior to reducing dimensions. ggplot2-based fviz_pca {factoextra} version 1.0.7 was used for graphical representation of individuals for the first two components.

For analysis, hierarchical clustering and heatmap visualization heatmap3 {heatmap3} version 1.1.9 was used. All missing values were removed (a total of 1601 proteins were used) and heatmaps were computed with and without reordering of columns. For imputing missing values, the imputerPCA {missMDA} was used with ncp = 2.

### 4.12. Statistical Analysis

The data were analyzed using GraphPad Prism (9.0 Mac OS X, USA) software, and all data were expressed as mean ± standard deviation (SD) with a statistical significance set at *p* < 0.05. For the proliferation assay and flow cytometry data, statistical significance was tested using two-way ANOVA and Sidak’s multiple comparison test. Mixed-effect model analysis with Tukey’s multiple comparison test was used on analysis of ICP data and qRT-PCR data. Finally, a Mann–Whitney *t*-test was used for comparison of fibroblast colony formation unit (CFU-F) frequencies and cell yield. Statistics used for proteomic data are outlined under mass spectrometry data analysis.

## Data Availability

Data will be available from the corresponding author on reasonable request and with permission of Odense University Hospital Legal Department, Odense, Denmark.

## Supplementary Materials

The following are available online at https://www.mdpi.com/article/10.3390/ijms23031692/s1.
