# Supplementary material for: Adipose-Derived Stem Cells from Type 2 Diabetic Rats Retain Positive Effects in a Rat Model of Erectile Dysfunction
Source: Int J Mol Sci. 2022 Feb 1;23(3):1692. doi: 10.3390/ijms23031692 (PMC8836282; doi:10.3390/ijms23031692)
Supplement: Supplementary file 1 [file ijms-23-01692-s001.zip › ijms-1500364-supplementary.pdf]

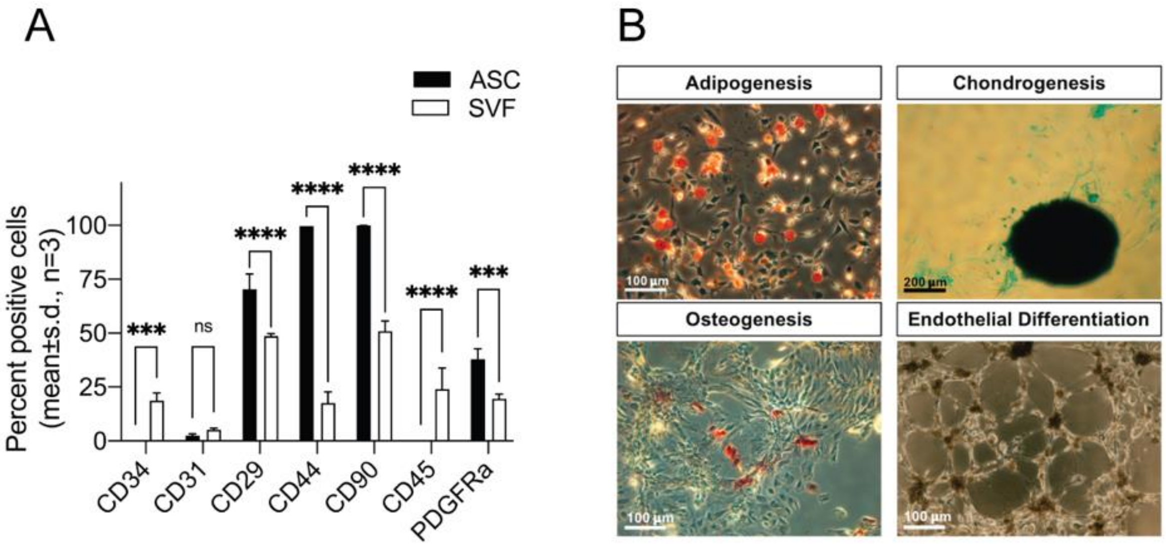

Figure S1. (**A**) The levels of mesenchymal stem cell markers of ASCs and SVF (white) cells were assessed by flow cytometry.and found to be significantly different between the two groups of cells. Data is shown as mean±SD, n=3 and statistical significance was tested using a Two-way ANOVA test with Sidak`s multiple comparison test. ***p<0.001, ****p<0.0001. (**B**) Confirmation of multipotency of ASCGK derived from inguinal fat was conducted by adipogenesis, chondrogenesis, osteogenesis and endothelial differentiation. Adipogenesis differentiation was verified by Oil Red O staining, chondrogenesis differentiation by Alcian Blue staining, osteogenesis differentiation by alkaline phosphatase detection and endothelial differentiation by tube-formation on Matrigel. Images were captured using an inverted microscope (Leica DMI4000B).

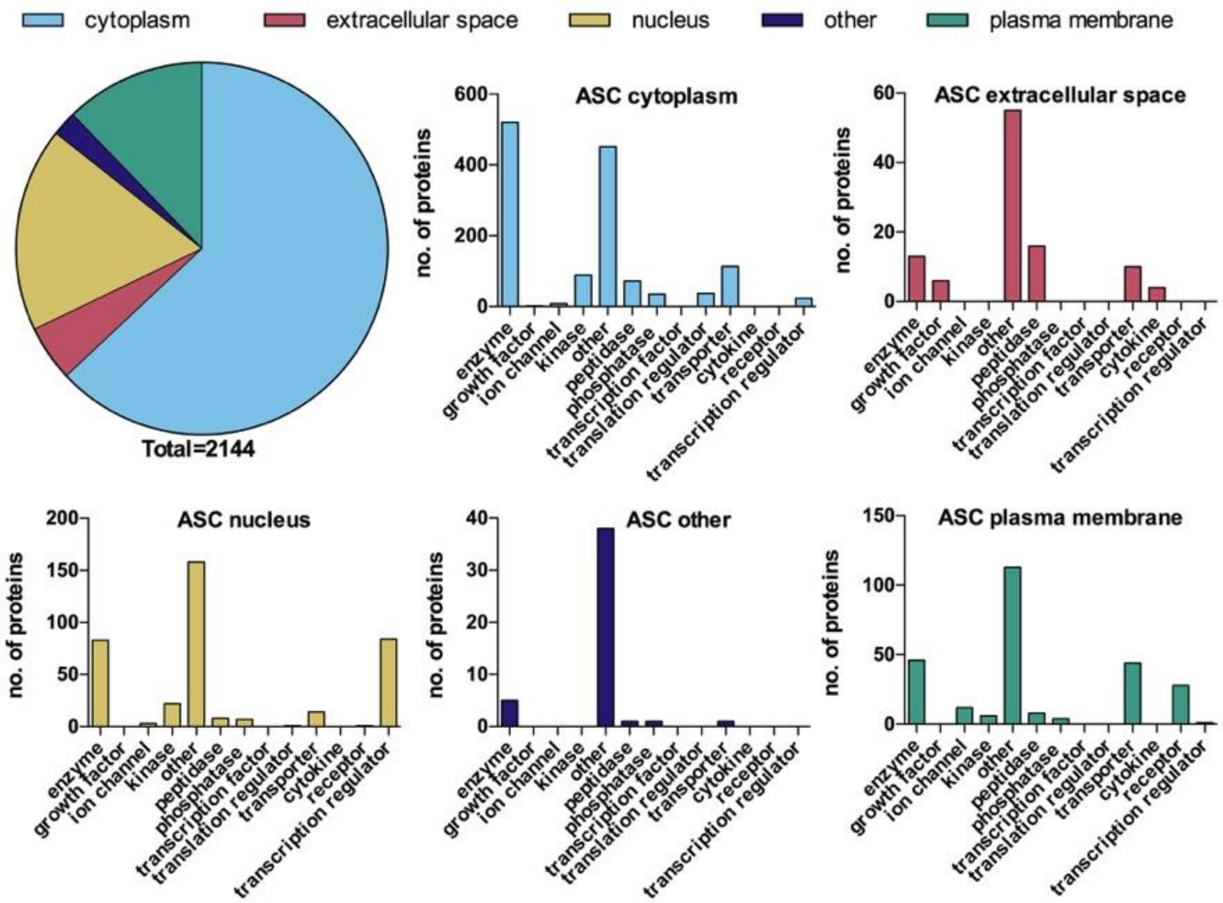

Figure S2. Classification of proteins identified in ASCs by TMT according to their subcellular localization and biological function. The classification was performed using the Ingenuity Pathway Analysis tool (IPA, Qiagen).

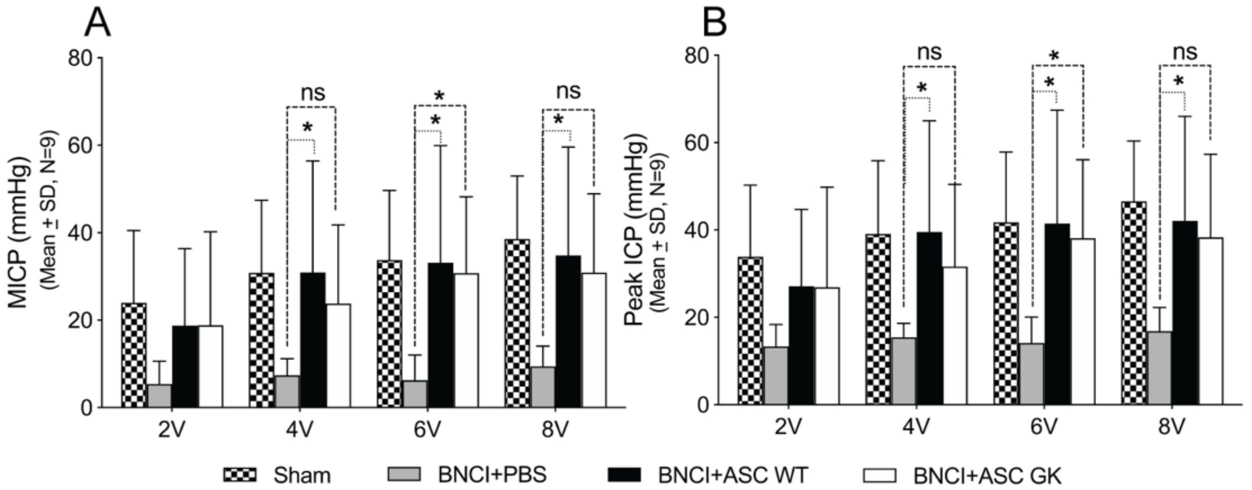

Figure S3. Rats were subjected to bilateral nerve crush injury (BNCI) followed by penile injection of vehicle (PBS) or ASC_{WT} or ASC_{GK}. 28 days after surgery the erectile function was evaluated by ICP (mmHg) recordings during electrostimulation of 2, 4, 6 or 8V. Several measures of erectile function were calculated: (**A**) the maximum ICP recorded during a stimulation was termed the peak ICP, (**B**) the baseline pressure in the crush was subtracted from the peak ICP to obtain the maximum ICP increase (MICP). Data are represented as mean±SD, and statistical significance.was tested by Mixed effect model (REML) with Tukey`s multiple comparison test. *p<0.05, ns- nonsignificant

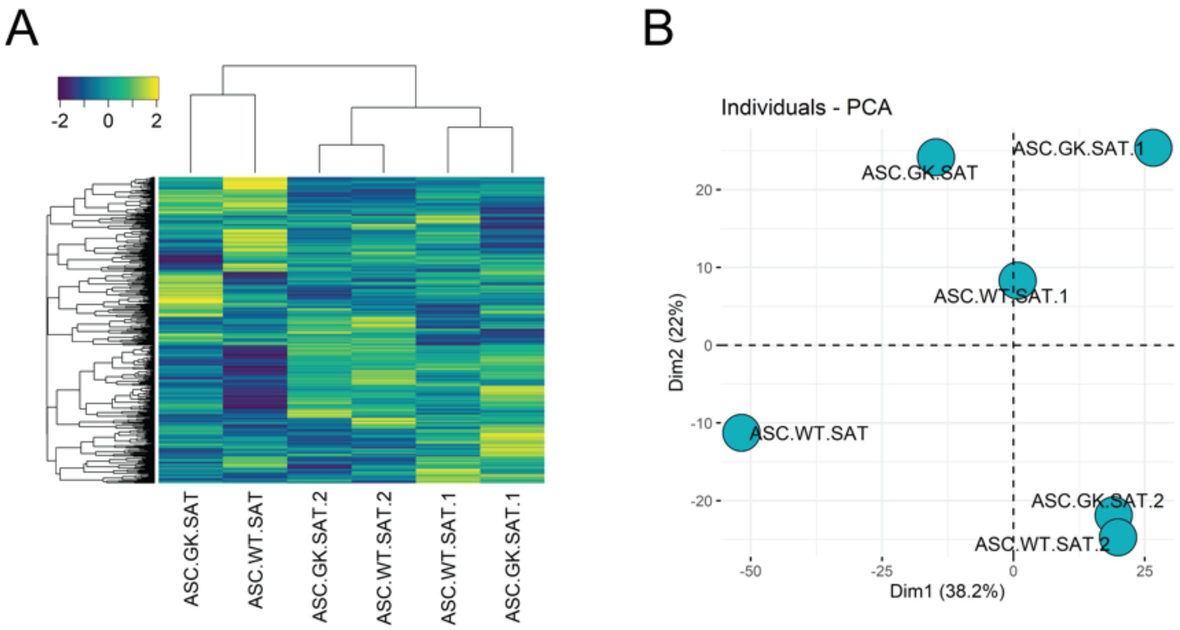

Figure S4. Cluster analysis of proteins with missing values were estimated using the R package missMDA. As for the analysis above, Heatmap (**A**) and PCA plot (**B**) did not show any difference between diabetic and non-diabetic ASCs.
